# The importance of adherence to international standards for depositing open data in public repositories

**DOI:** 10.1186/s13104-021-05817-z

**Published:** 2021-11-02

**Authors:** Diego A. Forero, Walter H. Curioso, George P. Patrinos

**Affiliations:** 1 Health and Sport Sciences Research Group, School of Health and Sport Sciences, Fundación Universitaria del Área Andina, Bogotá, Colombia; 2 Professional Program in Respiratory Therapy, School of Health and Sport Sciences, Fundación Universitaria del Área Andina, Bogotá, Colombia; 3 Vicerrectorado de Investigación, Universidad Continental, Lima, Peru; 4 Department of Pharmacy, School of Health Sciences, University of Patras, Patras, Greece; 5 Department of Pathology, College of Medicine and Health Sciences, United Arab Emirates University, Al-Ain, UAE; 6 Zayed Center for Health Sciences, United Arab Emirates University, Al-Ain, UAE

**Keywords:** Open science, Open data, Data repositories, Data reuse

## Abstract

There has been an important global interest in Open Science, which include open data and methods, in addition to open access publications. It has been proposed that public availability of raw data increases the value and the possibility of confirmation of scientific findings, in addition to the potential of reducing research waste. Availability of raw data in open repositories facilitates the adequate development of meta-analysis and the cumulative evaluation of evidence for specific topics. In this commentary, we discuss key elements about data sharing in open repositories and we invite researchers around the world to deposit their data in them.

## Introduction

There is an important global interest in Open Science, which include open data and methods, in addition to open access (OA) publications [1, 2]. Several funding agencies in the United States and in Europe have mandates for open data generated in the research projects they support. In addition, an increasing number of scientific journals have policies encouraging or asking authors to provide data in open repositories [3]. In this commentary, we discuss key elements about data sharing in open repositories, from an international and interdisciplinary perspective [4].

## Main text

### Open research data

It has been proposed that public availability of raw data increases their value and the possibility of confirming scientific findings, improving reproducibility and replicability of results [5–8], in addition to enhancing the options of reducing research waste [9]. In this context, the Transparency and Openness Promotion (TOP) guidelines promotes data transparency (https://www.cos.io/initiatives/top-guidelines) [7, 8]. It has been highlighted that there are several main types of research data repositories: Institutional, disciplinary, multidisciplinary and project specific [10]. Availability of raw data in open repositories facilitates the adequate development of meta-analysis, particularly individual patient data -IPD- meta analyses [11], and the cumulative evaluation of evidence for specific topics [12], especially for high-dimensional data [13] (such as results from genomics, transcriptomics or epigenomics). In this context, certain research fields, such as genomics, have developed standards that facilitate and promote deposition of raw data [14].

A recent study showed, in a sample of 531.889 OA journal articles, that a minor fraction of papers included a link to data repositories and that those articles have a higher citation impact [3]. Another recent work analyzed 487 papers describing clinical trials and found that, although many declared data availabilities, very few included data in repositories [15]. An analysis of 500 articles from 50 high-impact journals found that only a small fraction deposited their full raw data online [16]. In addition, in a sample of 49 published articles it was found that the reluctance to share data was associated with a weaker evidence and a higher number of errors in the reporting of statistical results [17]. Ioannidis and coworkers found that raw data unavailability led to a low rate of repeatability of microarray results from published articles [18].

The FAIR Guiding Principles have been proposed for scientific data management [19] and they involve these main four categories: Findable (unique and persistent identifiers, in addition to rich metadata), Accessible (retrievable by their identifier), Interoperable (a broadly applicable language for data representation) and Reusable (a clear and accessible usage license) [19]. Metadata, the information containing the details of data organization, collection and preprocessing, is key for the appropriate processes of finding, using and citing files in open repositories [20]. Recently, Corpas et al. have provided several recommendations to comply with the FAIR principles, such as establishing an adequate consent framework, maximizing machine-readable data and selecting the most findable and accessible data repositories [21]. Broman et al. have proposed several valuable recommendations for the organization of data files, such as being consistent, choosing adequate names for variables, avoiding empty cells, creating data dictionaries and using standard file formats (such as comma-delimited files) [22]. In this context, it has been shown that the use of some commercial file formats, such as.xls files, has led to issues in data storage, such as changing gene symbols to dates [23].

### Open access licenses and ethical aspects

There are several available OA licenses and the ones from Creative Commons (CC; https://creativecommons.org/about/cclicenses/) are frequently used [24]. CC BY is one of the less restrictive and involves attribution, CC BY-SA needs licensing under identical conditions, CC BY-ND does not allow derivative works, CC-BY-NC does not allow commercial uses and CC BY-ND-NC does not allow neither derivative works nor commercial uses [24]. It has been recommended [25] that a CC0 license (a universal public domain dedication; https://creativecommons.org/share-your-work/public-domain/cc0) should be used for data sharing.

There are several ethical aspects related to the sharing of data from human subjects, such as de-identification and having appropriate informed consents and approval by the institutional review boards [26–29]. In addition, in certain contexts, it is advisable the use of controlled-access repositories, in which the researchers need to apply to get access to the data. In specific cases of highly sensitive information, there is the option for the submission of processed data, such as summary statistics [25, 28]. The International Committee of Medical Journal Editors (ICMJE) requires, since 2017, that articles reporting the results of clinical trials should include a data sharing statement [30]. There are two major interesting examples of international sharing of data from patients and the development of important scientific findings and collaborations [28]: the Alzheimer’s Disease Neuroimaging Initiative (ADNI; adni.loni.usc.edu) has led to more than 2.100 international publications [31] and The Cancer Imaging Archive (TCIA; cancerimagingarchive.net) has facilitated the generation of more than 1.100 international publications [32]. In some regions of the world, there is the need for further training for members of research ethics committees about the multiple advantages of sharing data for the advancement of health sciences research [27, 28].

### Recommendations for researchers around the globe

In Table 1 we present a selection of major data repositories (some of them are for general use and others are oriented to specific applications or data types), in order to provide options to the readers to submit their raw results [25]. Among them, the databases at the National Center for Biotechnology Information (NCBI) contain several billion records; some of the largest databases from NCBI are the ones for DNA and RNA sequences (more than 429 million records), gene expression profiles (more than 128 million records), single nucleotide polymorphisms (SNPs; more than 720 million records) and protein sequences (more than 874 million records) [33]. Regarding the databases from the European Bioinformatics Institute, the largest resources are the European Nucleotide and Genome-Phenome Archives, the PRoteomics IDEntifications and the ArrayExpress [34]. The Protein Data Bank has more than 140.000 entries [35] and the Image Data Resource stores different types of imaging data [36]. DataMed (datamed.org) is a search engine for data deposited in repositories [37], there is the Registry of Research Data Repositories (re3data.org) [10] and the European Data Portal (https://data.europa.eu/en) facilitates consolidation and search of open datasets from that region of the world [38]. The Research Data Alliance (RDA) is an international initiative promoting multiple aspects related to open data sharing (https://www.rd-alliance.org) [39].

**Table 1:** Information about selected major open data repositories

Repository	URL	Type of data	Features
OSF	https://www.osf.io	All types	Individual files must be 5 GB or less
Zenodo	http://www.zenodo.org	All types	50 GB per dataset
Figshare	https://www.figshare.com	All types	File uploads of up to 5 TB in size
Dryad	https://www.datadryad.org	All types	A limit of 300 GB per data publication
NCBI GEO	https://www.ncbi.nlm.nih.gov/geo	Array- and sequence-based data	It encourages to supply MIAME- and MINSEQE-compliant data
ArrayExpress	https://www.ebi.ac.uk/arrayexpress	Array- and sequence-based data	It encourages to supply MIAME- and MINSEQE-compliant data
Image Data Resource	https://www.idr.openmicroscopy.org	Life sciences image data	For file sizes larger than 1000 GB special planning is needed
Protein Data Bank	https://www.rcsb.org	Atomic-level, 3D structure data	It uses the PDBx/mmCIF file format

Researchers can identify the repository with the highest affinity to their data type and needs of sharing, such as general repositories or platforms for specific types of data. The Registry of Research Data Repositories (re3data.org) provides a comprehensive list

There is a need for more training about open science and data science [25], particularly in emerging economies, and a larger number of open data repositories are very needed in these regions of the world [40, 41]. In this context, the adequate implementation of standards for reporting of raw data for specific fields, such as the MIAME (Minimum Information About a Microarray Experiment) [14], is key in order to provide an adequate organization of files and inclusion of key metadata, with information such as description of the individuals/samples, experimental conditions and analyses [20]. Funding agencies and academic institutions from multiple countries are invited to consider the importance of open data in their policies and incentives [41, 42]. Although it is a common practice in several journals, editors and peer reviewers of even more international publications should enforce the guidelines asking authors of manuscripts to deposit raw data [12] and scientists from around the world are invited to deposit their data in open repositories [20, 25, 43]. These efforts could be particularly catalyzed by initiatives such as microattribution [44, 45], which provides researchers incentives to openly share their data to the public domain, allowing not only open data sharing but also the possibility of reaching new scientific conclusions that would otherwise not be possible if these data are not being made publicly available [44]. Such initiatives have already been implemented for data repositories, such as locus-specific databases [44], national/ethnic mutation databases [46], clinical databases and consortia [47] and scientific journals (https://www.nature.com/sdata).

## Outlook

In times of COVID-19, it is critical to have good quality data (including aspects of accessibility, timeliness and support for users, among others [48]) for proper decision-making. We need data of high quality, that are reliable and trustworthy [49]. At the global level, initiatives like the Research Data Alliance COVID-19 Working Group involved 440 volunteer data experts to address several issues with data and software sharing to improve the response to the pandemic [49]. They provided recommendations and guidelines on data sharing [49].

However, several challenges have to be solved, particularly in emerging economies, such as: legal and policy issues, scarcity of coordination between research groups, lack of a culture for data sharing and ethical/privacy considerations, insufficiency of proper infrastructure (including high-speed Internet connectivity), deficiency in interoperability of platforms, shortage of data managers and data scientists and a scarcity of open data repositories to facilitate data sharing [50]. Recently, an examination of open government data portals for 60 countries found that USA, Czech Republic and Canada have the largest numbers of available datasets (more than 291,000, 136,000 and 85,000, respectively) [48]. In some cases, governments do not see the value for implementing open data repositories; besides it is an excellent way for transparency [48], accountability and even a strategy to deal with corruption. We all play a role in this pandemic, and we need more collaboration between private and public agencies, interdisciplinary approaches, universities, non-governmental organizations, and the civil society to promote an efficient use of open data repositories (as it has been demonstrated recently in the pandemic [51]). In addition, investing in health information systems, interoperability and incentives are key components. Governments should also monitor and evaluate the impact of sharing data on repositories. Finally, there is an important need to strength capacities in the biomedical personnel (particularly in emerging economies), in topics such as: data science, open data repositories, data intelligence, data protection regulations with multidisciplinary teams and collaboration between key stakeholders. As a very high number of publications about Open Science is written by authors from the Global North [8], it is needed to have more international articles about Open Data from the Global South [1, 4, 52].

## Data Availability

Not applicable.

## Abbreviations

CC: Creative Commons
MIAME: Minimum information about a microarray experiment
NCBI: National Center for Biotechnology Information
OA: Open access

## References

[1] Forero DA, Lopez-Leon S, Perry G (2020). A brief guide to the science and art of writing manuscripts in biomedicine. J Transl Med.

[2] Piwowar H, Priem J, Lariviere V, Alperin JP, Matthias L, Norlander B, Farley A, West J, Haustein S (2018). The state of OA: a large-scale analysis of the prevalence and impact of Open Access articles. PeerJ.

[3] Colavizza G, Hrynaszkiewicz I, Staden I, Whitaker K, McGillivray B (2020). The citation advantage of linking publications to research data. PLoS ONE.

[4] Onie S (2020). Redesign open science for Asia, Africa and Latin America. Nature.

[5] Hicks DJ (2021). Open science, the replication crisis, and environmental public health. Account Res.

[6] Allen C, Mehler DMA (2019). Open science challenges, benefits and tips in early career and beyond. PLoS Biol.

[7] Nosek BA, Alter G, Banks GC, Borsboom D, Bowman SD, Breckler SJ, Buck S, Chambers CD, Chin G, Christensen G (2015). Scientific Standards. Promoting an open research culture. Science.

[8] Munafo MR, Nosek BA, Bishop DVM, Button KS, Chambers CD, du Sert NP, Simonsohn U, Wagenmakers EJ, Ware JJ, Ioannidis JPA (2017). A manifesto for reproducible science. Nat Hum Behav.

[9] Ioannidis JP, Greenland S, Hlatky MA, Khoury MJ, Macleod MR, Moher D, Schulz KF, Tibshirani R (2014). Increasing value and reducing waste in research design, conduct, and analysis. Lancet.

[10] Pampel H, Vierkant P, Scholze F, Bertelmann R, Kindling M, Klump J, Goebelbecker HJ, Gundlach J, Schirmbacher P, Dierolf U (2013). Making research data repositories visible: the re3data.org Registry. PLoS One.

[11] Wang H, Chen Y, Lin Y, Abesig J, Wu IX, Tam W (2021). The methodological quality of individual participant data meta-analysis on intervention effects: systematic review. BMJ.

[12] Forero DA, Lopez-Leon S, Gonzalez-Giraldo Y, Bagos PG (2019). Ten simple rules for carrying out and writing meta-analyses. PLoS Comput Biol.

[13] Rung J, Brazma A (2013). Reuse of public genome-wide gene expression data. Nat Rev Genet.

[14] Brazma A, Hingamp P, Quackenbush J, Sherlock G, Spellman P, Stoeckert C, Aach J, Ansorge W, Ball CA, Causton HC (2001). Minimum information about a microarray experiment (MIAME)-toward standards for microarray data. Nat Genet.

[15] Danchev V, Min Y, Borghi J, Baiocchi M, Ioannidis JPA (2021). Evaluation of data sharing after implementation of the International Committee of Medical Journal Editors Data Sharing Statement Requirement. JAMA Netw Open.

[16] Alsheikh-Ali AA, Qureshi W, Al-Mallah MH, Ioannidis JP (2011). Public availability of published research data in high-impact journals. PLoS ONE.

[17] Wicherts JM, Bakker M, Molenaar D (2011). Willingness to share research data is related to the strength of the evidence and the quality of reporting of statistical results. PLoS ONE.

[18] Ioannidis JP, Allison DB, Ball CA, Coulibaly I, Cui X, Culhane AC, Falchi M, Furlanello C, Game L, Jurman G (2009). Repeatability of published microarray gene expression analyses. Nat Genet.

[19] Wilkinson MD, Dumontier M, Aalbersberg IJ, Appleton G, Axton M, Baak A, Blomberg N, Boiten JW, da Silva Santos LB, Bourne PE (2016). The FAIR Guiding Principles for scientific data management and stewardship. Sci Data.

[20] Michener WK (2015). Ten simple rules for creating a good data management plan. PLoS Comput Biol.

[21] Corpas M, Kovalevskaya NV, McMurray A, Nielsen FGG (2018). A FAIR guide for data providers to maximise sharing of human genomic data. PLoS Comput Biol.

[22] Broman KW (2018). Woo KHJTAS: data organization in spreadsheets. Am Stat.

[23] Ziemann M, Eren Y, El-Osta A (2016). Gene name errors are widespread in the scientific literature. Genome Biol.

[24] Carroll MW (2013). Creative commons and the openness of open access. N Engl J Med.

[25] Wilson SL, Way GP, Bittremieux W, Armache JP, Haendel MA, Hoffman MM (2021). Sharing biological data: why, when, and how. FEBS Lett.

[26] Meyer MN (2018). Practical tips for ethical data sharing. Adv Methods Pract Psychol Sci..

[27] Mello MM, Lieou V, Goodman SN (2018). Clinical trial participants' views of the risks and benefits of data sharing. N Engl J Med.

[28] Shahin MH, Bhattacharya S, Silva D, Kim S, Burton J, Podichetty J, Romero K, Conrado DJ (2020). Open data revolution in clinical research: opportunities and challenges. Clin Transl Sci.

[29] Cummings JA, Zagrodney JM, Day TE (2015). Impact of open data policies on consent to participate in human subjects research: discrepancies between participant action and reported concerns. PLoS ONE.

[30] Taichman DB, Sahni P, Pinborg A, Peiperl L, Laine C, James A, Hong ST, Haileamlak A, Gollogly L, Godlee F (2017). Data sharing statements for clinical trials: a requirement of the International Committee of Medical Journal Editors. PLoS Med.

[31] Jack CR, Bernstein MA, Fox NC, Thompson P, Alexander G, Harvey D, Borowski B, Britson PJ, Whitwell J, Ward C (2008). The Alzheimer's Disease Neuroimaging Initiative (ADNI): MRI methods. J Magn Reson Imaging.

[32] Clark K, Vendt B, Smith K, Freymann J, Kirby J, Koppel P, Moore S, Phillips S, Maffitt D, Pringle M (2013). The Cancer Imaging Archive (TCIA): maintaining and operating a public information repository. J Digit Imaging.

[33] Sayers EW, Beck J, Bolton EE, Bourexis D, Brister JR, Canese K, Comeau DC, Funk K, Kim S, Klimke W (2021). Database resources of the National Center for Biotechnology Information. Nucleic Acids Res.

[34] Cook CE, Stroe O, Cochrane G, Birney E, Apweiler R (2020). The European Bioinformatics Institute in 2020: building a global infrastructure of interconnected data resources for the life sciences. Nucleic Acids Res.

[35] ww PDBc (2019). Protein Data Bank: the single global archive for 3D macromolecular structure data. Nucleic Acids Res.

[36] Williams E, Moore J, Li SW, Rustici G, Tarkowska A, Chessel A, Leo S, Antal B, Ferguson RK, Sarkans U (2017). The image data resource: a bioimage data integration and publication platform. Nat Methods.

[37] Ohno-Machado L, Sansone SA, Alter G, Fore I, Grethe J, Xu H, Gonzalez-Beltran A, Rocca-Serra P, Gururaj AE, Bell E (2017). Finding useful data across multiple biomedical data repositories using DataMed. Nat Genet.

[38] Nikiforova A, McBride KJT (2021). Informatics: open government data portal usability: a user-centred usability analysis of 41 open government data portals. Telematics Inform..

[39] Treloar AJ (2014). The research data alliance: globally co-ordinated action against barriers to data publishing and sharing. Learn Publ.

[40] Fenner M, Crosas M, Grethe JS, Kennedy D, Hermjakob H, Rocca-Serra P, Durand G, Berjon R, Karcher S, Martone M (2019). A data citation roadmap for scholarly data repositories. Sci Data.

[41] Perrier L, Blondal E, MacDonald H (2020). The views, perspectives, and experiences of academic researchers with data sharing and reuse: a meta-synthesis. PLoS ONE.

[42] Demetres MR, Delgado D, Wright DN (2020). The impact of institutional repositories: a systematic review. J Med Libr Assoc.

[43] Figueiredo AS (2017). Data sharing: convert challenges into opportunities. Front Public Health.

[44] Giardine B, Borg J, Higgs DR, Peterson KR, Philipsen S, Maglott D, Singleton BK, Anstee DJ, Basak AN, Clark B (2011). Systematic documentation and analysis of human genetic variation in hemoglobinopathies using the microattribution approach. Nat Genet.

[45] Patrinos GP, Cooper DN, van Mulligen E, Gkantouna V, Tzimas G, Tatum Z, Schultes E, Roos M, Mons B (2012). Microattribution and nanopublication as means to incentivize the placement of human genome variation data into the public domain. Hum Mutat.

[46] Georgitsi M, Viennas E, Gkantouna V, Christodoulopoulou E, Zagoriti Z, Tafrali C, Ntellos F, Giannakopoulou O, Boulakou A, Vlahopoulou P (2011). Population-specific documentation of pharmacogenomic markers and their allelic frequencies in FINDbase. Pharmacogenomics.

[47] Sosnay PR, Siklosi KR, Van Goor F, Kaniecki K, Yu H, Sharma N, Ramalho AS, Amaral MD, Dorfman R, Zielenski J (2013). Defining the disease liability of variants in the cystic fibrosis transmembrane conductance regulator gene. Nat Genet.

[48] Nikiforova A (2021). Smarter Open Government Data for Society 5.0: are your open data smart enough?. Sensors.

[49] Callaghan S (2020). Data sharing in a time of pandemic. Patterns.

[50] Curioso WH, Carrasco-Escobar G (2020). Collaboration in times of COVID-19: the urgent need for open-data sharing in Latin America. BMJ Health Care Inform.

[51] Xu B, Gutierrez B, Mekaru S, Sewalk K, Goodwin L, Loskill A, Cohn EL, Hswen Y, Hill SC, Cobo MM (2020). Epidemiological data from the COVID-19 outbreak, real-time case information. Sci Data.

[52] Adetula A, Forscher PS, Basnight-Brown D, Azouaghe S, Ouherrou N, Charyate A, Hansen N, Adetula GA. IJzerman H. Synergy between the credibility revolution and human development in Africa. 2021. 10.31730/osf.io/e57bq.

